# Lemnaceae as a poultry feed supplement: a review on the nutritional and economic potential for long term feed sustainability

**DOI:** 10.1186/s40104-026-01415-w

**Published:** 2026-05-16

**Authors:** Aakarsh Salian, Manikprabhu Dhanorkar, Satish Tongaonkar, Dibyendu Dey, Sarwar Ali, Piruthiviraj Kumar, Pooja Singh

**Affiliations:** 1 Symbiosis Centre for Waste Resource Management, Symbiosis International (Deemed University), Lavale, Pune, India; 2 Immeureka Animal Health Pvt. Ltd., Secunderabad, Telangana India

**Keywords:** Duckweed, Feed sustainability, Protein source, Poultry, Soybean

## Abstract

Soybean is a conventional and widely used protein source in the poultry feed. Fluctuating soybean prices linked to changing climatic and agroeconomic condition brings in multiple sustainability and food security concerns in the poultry feed sector. Additionally, competition for agricultural land, coupled to rising feed costs has put economic strain on the consumers. There is a need to promote alternative low-cost protein sources to bring in circularity in production systems, reduce environmental footprints, and improve long-term resilience of the poultry feed sector in the face of climate change. This work explores duckweed as a promising and economical alternative to soybean for use in the poultry feed sector and evaluates the limitations in their widespread use. Rich protein (around 35%–40%), high lysine content and diverse nutritional profile of duckweed confirm its suitability as an attractive component in the poultry feed, particularly relevant in the era of climate change and feed security crisis. Large scale production is however limited by processing and storage concerns that need to be further addressed to increase the economic viability of duckweeds. Production systems based on recycled wastewater makes the process sustainable. Reduction in antinutritive factors by enzymes or microbial fermentation will further enhance the acceptability of these plants as feed components. This study highlights opportunities to reduce the dependency on climate-sensitive soybean while promoting a circular bioeconomy approach in the poultry feed sector. Use of duckweed offers a promising approach to ensure a climate resilient and sustainable poultry sector, with potential to enhance food security.

## Introduction

Increasing world population has caused global food scarcity which is further exacerbated by climate change and resource depletion. Poultry consumption has increased in recent years, primarily due to increased health awareness and high protein content of poultry meat [1]. Global poultry feed market was valued at around USD 222.94 billion in 2025 and is projected to increase to USD 273.85 billion in 2034 [2]. Indian poultry feed market is expected to increase from USD 18.0 billion in 2025 to 25.25 billion by 2030 [3]. Increase in the demand of poultry products necessitates strategic interventions to ensure the availability of affordable poultry feed. However, climate change is reshaping global agricultural systems and influencing food availability, including the components used in poultry feed. Nutritional quality and production costs in poultry farming is deeply impacted by changing climate conditions and fluctuating food market. Strategic interventions to bring in circularity and sustainability in the poultry feed sector are hence imperative.

Protein forms a vital part of poultry feed composition, making the cost of protein source a primary variable shaping the overall cost of poultry feed products. Conventionally soybean meal and fish meal are the two primary sources of protein used extensively in poultry feed [4]. From an agronomic aspect, conventional poultry feed like soybean and alfalfa, require large agricultural lands and have a huge environmental footprint. Additionally, depleting ground water, erratic weather patterns, soil degradation, land use competition and fluctuating supply chain cause steep rise in prices of soybean and other feed ingredients, sharply impacting the poultry sector that relies heavily on soybean as primary protein source [5]. Hence a more stable and economical alternative is needed to ensure sustainability in this sector. There is a growing interest and need to explore and establish alternate, diverse, and sustainable protein sources for incorporation in poultry feed. Alternate and low-cost protein sources will not only help maintain a steady supply of a vital feed ingredient, but also ensure cost efficiency of the feed, a critical aspect in the poultry industry. Diversifying protein sources will bring stability in feed production and use of locally available low-cost biomass can ensure sustainability [6]. Using non-food sources as poultry feed component will reduce the competition for staple crops that are parallelly also used for human consumption.

Insect meal [7], animal by-products [8], plant-based by-products [9] and fermented agricultural waste [10] have shown promise for use as an alternate protein source in poultry feed. Another low cost, readily available and highly nutritious wholesome source of protein is the aquatic plant duckweed. Duckweed is free floating aquatic plant, found in fresh-water bodies across many tropical and sub-tropical regions of the world. They are the smallest flowering plants with a high protein and micronutrient content, known in South-East Asian region as “Kai hin” or “Khou nam” [11]. Positive effects on fishes, humans and animals have been reported upon duckweed consumption and many commercial products are present in the mainstream market [5, 12, 13]. Despite its rich protein content, fast growth, favourable nutrient profile, and ecological significance, duckweed remains underexplored in mainstream poultry nutrition.

The aim of the current work is to evaluate the potential of duckweed as an alternative source of protein in poultry feed as a sustainable and low-cost feed additive, especially in the face of changing environmental conditions and resource depletion. The nutritional composition of duckweed, effect of feed water on nutritional profile, inclusion characteristics, metabolic advantages, and environmental benefits are especially addressed in this work. This work is a scoping review that critically examines the above variables, identifies current industry limitations, and proposes potential directions for future research in the use of duckweed as a poultry feed supplement in the era of climate change. Online scientific databases, including Scopus, Google Scholar and Web of Science were used to collect comprehensive data from previous research on duckweeds. The search incorporated combinations of keywords such as *“duckweed”*,* “Lemna”*,* “poultry nutrition”*,* “poultry feed”*,* “antinutritional factors”*, *“sustainable feed systems”*, “*amino acid profile*”, and “*cultivation*”. Boolean operators (AND, OR) were used to refine the search. Peer reviewed articles published in English were included to extract relevant data on duckweed cultivation, their exploration as poultry feed, nutritional profile, metabolic responses on poultry and other relevant information. Challenges and limitation were then critically identified and examined from the published reports to highlight potential areas for future investigation.

While duckweed nutritional prowess is well established, the specific influence of water quality on the nutritional profile of duckweed, and a focused perspective on the effect of duckweed inclusion in poultry feed with practical feed inclusion data is lacking. By addressing these barriers, researchers and stakeholders can better assess the viability of duckweed as a sustainable, cost-effective, and nutritionally balanced feed supplement for poultry. By evaluating duckweed through a poultry specific lens and highlighting its role in low-cost sustainable feed systems, we aim to provide stakeholders with a novel perspective towards practical integration of duckweed into climate-resilient cost effective poultry feed production systems and integration with water stewardship in the region.

## Duckweed: Tiny power packed plants as alternative to traditional protein

Duckweed is a monocot, flowering aquatic plant, also known as ‘water lentils’. Duckweed is a free-floating plant which belongs to the family of Lemnaceae, consisting of two sub families Lemnoidea and Wolffioidae represented by five genera, *Spirodela, Lemna*, *Wolffia, Landoltia,* and *Wolffiella* comprising of 34 species [14]. Duckweed plants measure between 0.4 to 15 mm in size and are also known to be the smallest flowering plants in the world with no true leaves or true stem. The leaves are composed of specialized collenchymous cells called aerenchyma which are enclosed large intercellular spaces that create a buoyancy in the plant and help in floatation [15]. Duckweed plants consist of under differentiated fronds with an attached leaf and stem, propagating through asexual budding or vegetative reproduction. Duckweed is fast growing and easy to cultivate plant that grows well on slow flowing or stagnant water enabling it to be easily grown on farms at a low cost.

Duckweed as a poultry feed ingredient is fast attracting global attention, primarily due to its nutritional composition and ecological significance. The nutritional composition of *Lemna minor* is reported by many groups, underscoring the critical importance of duckweed as an alternate source of protein (Table 1).

**Table 1:** Comparative nutritional composition of various feed ingredients used in poultry farming (expressed as % of dry matter)

Feed ingredient	Dry matter, %	Crude protein, %	Ether extract, %	Crude fibre, %	Ash, %	References
Other protein sources						
Feather meal	93.2	90.4	8.4	0.7	1.3	[16]
Fishmeal	93.1	65.1	6.1	0.3	26.5	
Soybean meal (47% Crude protein)	90.3	52.2	2.4	2.8	7.7	
Soybean meal (44% crude protein)	93.3	46.8	1.4	5.4	7.3	
Rapeseed meal	93.4	40.8	1.4	7.9	10.9	
Full-fat Soybean	94.8	38.6	18.2	4.07	6.06	
Skim milk	97.0	32.9	1.1	ND	7.6	
Brewer’s grain	94.3	31.4	6.4	11.6	3.8	
Corn DDGS	91.9	30.7	10.2	8.2	5.6	
Energy Sources						
Broken rice	89.4	9.5	1.5	1.4	0.3	
Corn	89.9	9.0	5.6	1.7	1.3	
Rice bran	92.9	16.1	20.6	9.2	11.2	
Coconut meal	93.5	13.9	20.4	17.8	4.2	
Other feed components						
Palm kernel meal	94.9	16.08	11.171	18.1	4.6	
Soybean hull	92.6	12.3	3.4	44.9	4.5	
Duckweed as protein source						
*Lemna*	91.5	36.8	5.2	10.9	14.206	
*Wolffia*	95.3	44.3	1.7	6.3	14.934	
*Spirodela*	93.5	17.96	4.8	17.06	21.2	
*Lemna*	6.7	27.4	4.3	10.37	12.26	[17]
*Lemna*	NA	38	3	16.16	14.6	[18]
*Lemna*	92	31.7	3.2	8.7	3.8	[19]
*Lemna*	91.7	28.1	7.9	7.7	8.2	[20]
*Spirodela*	NA	30.5	1.9	17.0	9.5	[21]
*Lemna*	7.9	40.2	7.9	12.3	14.0	[22]
*Lemna*	7.0	39.1	7.7	12.3	14.7	[22]

*DDGS* Distillers dried grains with solubles, *NA* Not available, *ND* Not detected

*Lemna*, *Wolffia* and *Spirodela* species of duckweed have been primarily explored for their potential as a poultry feed component (Table 1). Protein concentration ranging from around minimum 18% to maximum 44% have been reported from many duckweed species studied till date, as is also evident from Table 1. The crude protein content of duckweed is influenced by species and nutrient availability in the growth medium. Nevertheless, duckweed amino acid profile is diverse and rich with asparagine, aspartic acid and glutamic acids most abundant amino acids, while methionine, tryptophan and cysteine are reported in lowest concentrations (Table 2).

**Table 2:** Amino acid profile of selected duckweed species

Duckweed species	*Spirodela polyrhiza*	*Landoltia punctata*	*Lemna minor*	*Lemna gibba*	*Wolffia globosa*
Amino acid, g/100 g protein					
Lysine	1.1–7.5	2.9–20.2	1.6–6.9	3.5–7.1	5.0–5.6
Leucine	0.1–9.1	0.3–7.3	0.4–8.4	6.0–9.4	6.9–7.7
Isoleucine	0.03–5.0	0.06–3.5	2.2–4.5	3.0–4.7	3.3–3.8
Valine	0.4–6.4	0.9–4.6	2.1–5.6	3.2–6.8	4.3–4.9
Phenylalanine	0.04–5.9	0.1–4.5	2.5–5.0	3.6–7.2	4.2–4.9
Threonine	1.02–22.8	2.5–17.5	0.1–4.8	2.7–4.9	3.9–4.5
Methionine	0.01–1.6	0.09–1.6	0.1–1.8	0.8–2.6	1.5–1.7
Histidine	0.1–6.4	0.4–5.3	0.3–2.3	1.4–2.8	1.6–2.0
Tryptophan	0.01–1.5	0.02–1.5	0.1–1.4	0.7–1.2	0.8–1.4
Alanine	0.06–28.1	4.0–5.3	1.2–5.9	4.0–6.2	6.4–6.9
Glycine	0.3–12.8	0.7–17.7	0.2–5.5	2.9–5.8	4.7–5.5
Serine	0.4–18.9	1.0–17.1	2.9–5.1	2.7–4.8	4.2–5.0
Proline	0.4–5.3	0.9–4.1	1.2–4.9	2.5–5.3	4.0–4.6
Arginine	0.9–12.4	2.7–11.9	3.1–9.3	3.3–7.2	5.6–5.8
Tyrosine	0.08–4.5	1.0–4.7	1.3–4.4	2.0–5.7	3.1–3.5
Aspartic acid	0.09–22.5	0.2–21.8	6.7–18.9	6.2–11.6	9.7–14.0
Glutamic acid	1.7–14.7	1.1–9.5	5.2–12.5	8.1–14.3	10.8–11.1
Cysteine	0.7–1.2	0.01–1.1	0.1–1.3	0.2–1.1	1.5–1.6
Asparagine	0.3–29.6	2.6–31.6	2.1–3.1	2.0–2.8	2.1–3.0
Glutamine	0.1–26.9	0.3–13.4	3.6–4.6	3.4–4.3	3.6–4.5
References	[23–26]	[24, 25]	[23, 25–28]	[23, 25, 26]	[30]

Duckweed is generally rich in essential amino acids that are present in higher concentrations, except methionine and cysteine (Table 2). Additionally, the amino acid profile of *Lemna* sp. is closer in composition to animal protein making World Bank and the Food and Agricultural Organization (FAO) recommend *Lemna* species as part of feed for fish, poultry and cattle [6, 23]. Duckweed has comparatively more lysine than any other protein source used in poultry feed, containing on an average around 4–5 g lysine per 100 g protein. [23, 25, 29]. Dry matter and crude protein provide baseline for nutrient comparison. The higher the crude protein, the lower the digestibility and available energy. Crude fibre provides structural carbohydrates such as cellulose and lignin, while ash content provides mineral content, supplying essential macro- and micronutrients. However, excessive ash dilutes energy and protein while also lowering the digestibility of the feed.

Ether extract (EE) represents fraction of fats, oil and other related compounds. Higher EE content is advantageous for energy-dense diets, especially for monogastric animals [15]. Fat content in different duckweed species studied till date ranges from around 3%–17%, with polyunsaturated fatty acids, arachidonic acid, palmitic acid, linoleic acid and linolenic acid, and palmitic acid from among saturated fatty acids, found in abundance [18, 20, 31–33]. Presence of high amounts of short chain fatty acids (16%) indicates a longer shelf life for duckweed biomass, as short chain fatty acids are known to be bacteriostatic [34]. Fatty acid composition also varies with species, with *Lemna minor* reporting a higher proportion of linolenic acid as compared to *Spirodela polyrhiza* [35].

Duckweed is also rich in multiple secondary metabolites, such as carotenoids, especially lutein and beta carotene, that support eye health and help reduce the risk of chronic heart diseases. Abundance of xanthophyll, carotene, and other flavonoids makes duckweed a rich source of antioxidants and anti-inflammatory compounds [25]. Additionally, duckweed contains multiple essential minerals (~0.2%–7%), including potassium (K), iron (Fe), calcium (Ca), and phosphorus (P), along with trace elements such as manganese (Mn), zinc (Zn), and copper (Cu), which are important for various metabolic processes [30, 36]. This is a critical feature that, although beneficial, needs monitoring, as accumulation of toxic heavy metals from the growing water can prove to be detrimental for the receiving consumer. Apart from vitamin C and E, duckweed biomass is reported to contain around 0.5 and 10 μg per 100 g dry matter of Vitamin B_12_. This is possibly originating from the duckweed associated bacteria, underscoring the importance of plant-water environment in modulating the nutritional profile of duckweed [37].

Protein, fibre, and ash contents vary with duckweed species and feed water [16]. These in turn influence the digestibility outcomes of duckweed. Duckweed species like *Lemna minor* and *Lemna gibba* typically show higher digestibility compared to *Spirodela polyrhiza*, which in comparison to other duckweed species has higher fibre content [33]. Presence of a complete amino acid profile, vitamins, minerals, and antioxidants, and high amounts of polyunsaturated fatty acids make duckweed a superior food additive as compared to other protein sources, including soybean [16]. Along with a rich and compatible nutritional profile, duckweed can be a highly customizable and nutritionally diverse functional feed ingredient, capable of being "grown to specification" depending on the nutritional needs of the target livestock. This level of nutritional control is not typically possible with conventional feed crops like soybean, which are more reliant on fixed soil and climate conditions [16].

## Duckweed as poultry feed: evidence from studies

Multiple studies in the last decade were focused on making duckweed an integral part of poultry feed sector. Multiple and diverse effects on poultry growth and wellbeing have been reported in these studies from no effect on growth, to an increase in weight gain up to a limited level of duckweed inclusion (Table 3).

**Table 3:** Synergistic effects of duckweed when used as a supplement in mixed rations for poultry

Sr. No.	Feed variations	Type of poultry	Results	References
1	Duckweed replaced fish-meal (up to 6%)	Growing ducklings	Performance comparable to control	[38]
2	0, 5% and 10% *L. minor*, with and without enzyme	Broiler chicken	Increase in growth rate was observed in control and 5% *L. minor* with enzyme	[39]
3	Fermented and non-fermented duckweed (*Lemna*) added to basal feed	Free range chicken	Superior weight gain compared to control	[40]
4	0–25% inclusion of *Lemna gibba*	Titan Broiler chicken	Weight gain and egg production till 15% inclusion similar to control; 25% inclusion caused decrease in feed consumption and weight gain	[41]
5	0–40% inclusion of *Lemna gibba*	Leghorn layer hens	No change on egg production or egg weight; 15% inclusion caused higher protein and yolk pigmentation in eggs	[42]
6	Duckweed replaced fish-meal (up to15%)	Laying hen	Performance comparable to control; Feed conversion ratio (FCR) and egg pigment improved	[43]
7	Three different feeds assessed: Rice bran + Soybean, Rice bran + high content protein (*L. minor*), Rice bran + low content protein (*L. minor*)	Ducks	There was an increase in weight and improvement of skin tone of ducks by feeding the ducks with *L. minor* feed	[44]
8	Diet with and without fresh duckweed (no replacement of any component)	Starter chicks	Higher weight gain and faster appearance of feathers	[45]
9	Ducks were fed with different ratio of *L. minor,* water spinach and broken rice feed	Ducks	There was higher growth of ducks and FCR when fed with *L. minor* and lower growth rate when fed with water spinach	[46]
10	Sesame oil cake replaced with *L. minor*	Seven-day old Vencobb commercial broiler chicks	Partial replacement with *L. minor* showed marginal increase in the growth rate of chicks	[47]

Empirical studies demonstrate that incorporating dried duckweed up to 10%–12% in broiler diets and 15%–20% in layer feeds can maintain optimal growth, feed conversion, and egg quality [9], although caution is advocated against exceeding 6% inclusion without further studies [48]. In other report, replacement of fish meal up to 25% at starter phase and 50% at finisher phase is recommended without any negative effects [49]. The proportion of duckweed replacing traditional feed may affect the digestibility and absorption of nutrients by animals and hence thorough trials and studies are needed before duckweed is incorporated into poultry diets.

**Fig. 1:**
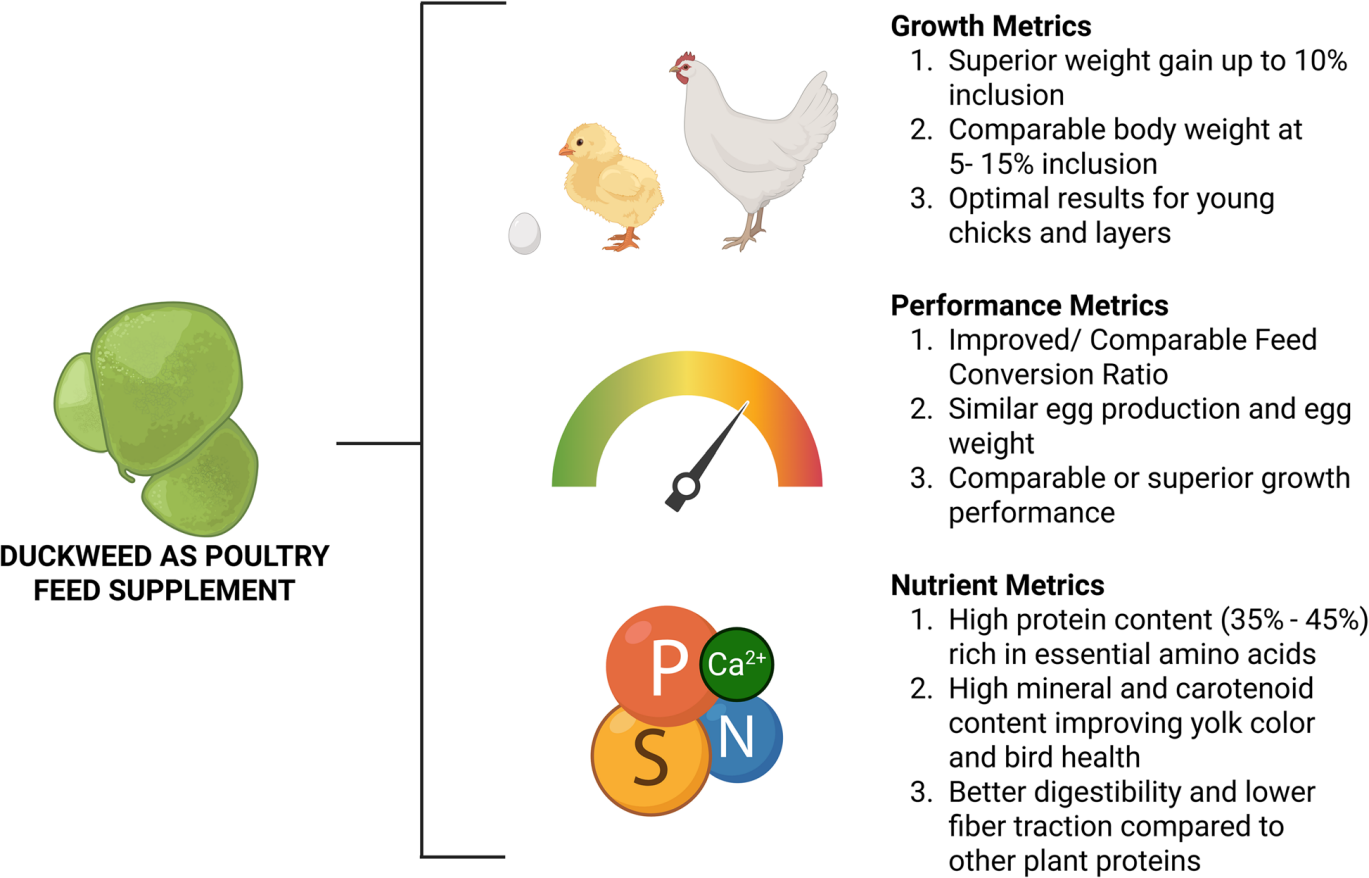
Performance of duckweed as poultry feed supplement (created in https://BioRender.com)

The effects of duckweed feed on poultry vary from the impact on performance metrics to a longer impact on gut health and immunological response of the poultry receiving duckweed supplemented feed (Fig. 1).

### Performance metrics: growth rate, weight gain and feed conversion efficiency in poultry

Performance metrics are important for poultry production and include parameters such as growth rate, weight gain and feed conversion efficiency. Growth rate measures increase in body weight of chicken and weight gain is referred in terms of daily increase in body weight. Feed conversion efficiency is assessed by feed conversion ratio (FCR), which refers to the amount of feed required for each unit of weight gain. Hence, the lower the FCR, the better the feed efficiency [50]. It has been earlier reported that duckweed at a maximum inclusion level of 10%–12% can be given to chick starter or broiler without negatively affecting weight gain and feed efficiency [39, 51]. It will be an interesting line of study to assess the FCR for different species of *Lemna* or *Spirodela* in single or polyculture use.

### Impact on egg production and meat quality

Duckweed inclusion in poultry feed impacts egg laying capacity of chicken but contrasting reports are available on this, varying with the type and body mass of the poultry. Many reports indicate a decrease in the egg laying rate of poultry fed with duckweed supplemented poultry diet. This decrease can be attributed primarily to the presence of indigestible crude fibre in duckweed, that in turn varies with the species of the duckweed used [38, 52]. Inclusion of duckweed in poultry diet increased yolk pigmentation in the egg, possibly due to the presence of carotenoids, including β-carotene, α-carotene, violaxanthin, zeaxanthin, lutein in duckweed. Additionally, blood spots in egg increased by 20% and dry albumin decreased by 10% as compared to the control feed [38, 52].

There are contrary reports that indicate a positive effect of duckweed inclusion in poultry feed on egg production and quality, when used in moderate supplementation. Duckweed inclusion in diet influences egg quality by a mix of factors including nutrient enrichment, pigment deposition and antinutritional interactions. These are attributed to the rich nutritional profile of duckweed that enables accumulation of healthy and acceptable proteins and lipid content in eggs. Duckweed was found to be a good source of protein for laying hens showing no adverse effect on production performance till an inclusion level 130 g/kg and no negative effect on egg quality characteristics when used up to 150 g/kg level [43]. Replacement of inorganic salts with dried duckweed enriched with trace elements in diets of Lohmann Brown layers proved beneficial and enhanced egg quality parameters [53]. Use of duckweed to replace sesame oil cake in feed of broilers at high concentrations were not much encouraging but improvement in body weight was observed at lower levels (3%–6%) of duckweed supplementation [47]. Inclusion of duckweed in laying hens fed with duckweed at a rate of 12.6% enhanced omega-3 levels in eggs, but no significant effect on egg production was observed [54]. An increase in protein and yolk pigment content of eggs was reported when duckweed was included at a rate of 15%–25% in feed of laying Leghorn hens [42]. There was no impact on egg production or egg weight in duckweed and non-duckweed fed hens. Similar increase in egg pigmentation was reported in another report wherein increase in liver enzymes aspartate aminotransferase and alanine aminotransferase was observed in duckweed fed layer hens, suggesting additional hepatoprotective effect of duckweed supplemented feed [55].

### Digestibility and nutrient absorption

As a feed additive for cattle and poultry, duckweed was observed for its remarkable digestibility and nutrient absorption efficiency. Duckweed has less lignin and cellulose than other forages, which improves enzymatic access to proteins and carbohydrates, and increases total digestibility for ruminant and monogastric species [56]. There have been studies wherein the fibre content in duckweed (18.06%) caused a reduction in the absorption of nutrients as compared to the conventional feed soybean. This effect can be overcome by the supplementation of duckweed with digestive enzymes, such as phytase, that could increase the absorption of nutrients leading to easy digestibility [39]. Duckweed's balanced amino acid profile, containing lysine, methionine, and threonine, amino acids that are sometimes limiting in diets based on cereals, is closely related to the efficiency of nutrient absorption in the animals receiving the feed. Research on chickens has demonstrated that adding *Lemna gibba* and *Spirodela polyrhiza* at moderate levels (up to 15%) can enhance egg protein content and yolk pigmentation while maintaining normal feed intake and growth performance [39, 52, 57].

Although nutritionally rich, at greater inclusion levels, palatability and absorption of duckweed rich feed can be influenced by excessive mineral or anti-nutritional elements such as phenolics and oxalates [11]. Additionally, digestibility of duckweed also varies between different species and on the growing environmental conditions. Compared to species grown in water logged land or shallow ponds, duckweed cultivated in nutrient-rich wastewater has lower crude fibre and higher crude protein levels (25%–45% dry matter), that enhances the amount of digestible protein [58]. High concentration of tannins in duckweed can bind to proteins and significantly reduce the standardized ileal digestibility of essential amino acids like methionine and cysteine, which reduces muscle development in chicks. Additionally high-water binding capacity of duckweed fibre can cause swelling in the gut and a sensation of satiety in chicks which can lead to reduced diet and reduced body weight [59].

## Duckweed cultivation for sustainability in poultry feed sector

There have been many advancements in the poultry feed sector over the last decade with work being targeted to enhance poultry feed efficiency and productions using analytical and predictive softwares [60]. In the last decade, focus in the poultry sector is to address the high feed costs through alternative feed ingredients, improve disease control, biosecurity measures to mitigate frequent outbreaks, and managing welfare issues such as the shifts away from conventional cages in layer farms*.* Efforts in improving cold chain and transportation infrastructure, ensuring food safety compliance for international trade, develop more efficient market access, investments in training, scientific research, and policy implementation have gained importance for sustained growth and competitiveness in the poultry industry [61]. Duckweed could be an alternative key player and a game changer in advancing the poultry feed sector, with many aspects making duckweed an attractive next gen poultry feed additive. With the potential to maximise profitability, meet nutritional needs more accurately, and reduce environmental pollution for more sustainable poultry production, duckweed is an promising player in the poultry industry [62].

### Cost-effectiveness of duckweed cultivation and biomass processing

Duckweed has a higher growth rate estimated at an average annual yield of around 30 tons per hectare per year with an annual revenue of $20,000–30,000 per hectare [63, 64]. Soybean cultivation, on the other hand, yields around 2.5–3.0 tons per hectare with a revenue of $1,000–$1,800 per hectare. Duckweed removes 99% of nutrients from wastewater, including nitrogen, phosphorus, and heavy metals, contributing parallelly to carbon fixation, thereby contributing to reduction in greenhouse gas emissions. Duckweed cultivation requires minimal fertilizers, pesticides and land space compared to other traditional poultry protein and can be harvested multiple times per month ensuring high profitability [65]. Additionally, the dual role of duckweed in wastewater remediation enhances its value as a sustainable and eco-efficient feed resource for the poultry industry.

### Environmental sustainability and resource efficiency

Cultivation of duckweed requires minimal land. Small artificial water bodies with wastewater can also be utilized for duckweed growth and cultivation making the process economical and environmentally sustainable [58, 66]. Digested slurry from biogas plant, wastewater from dairy industry, swine wastewater, and domestic wastewater have all been explored for duckweed cultivation, with nitrogen (N) and phosphorous (P) content playing a critical role (Table 4).

**Table 4:** Comparison of yield and water quality parameters for various duckweed species

Sr. No.	Duckweed	Water type	Growth rate	COD, mg/L	N, mg/L	P, mg/L	Reference
1	*Lemna minuta*	Dairy farm digested slurry	3.1 g/m^2^/d (dry weight)	NA	13.7	1.6	[36]
2	*Lemna minor*	Pond supplemented with organic manure	70.25 g/m^2^/month (dry weight)	NA	3.3 (NH_3_-N)	1.5	[58]
3	*Lemna Minor*	Synthetic leachate	7 g/m^2^/d	1,571	46.6	42.7	[67]
4	*Landoltia punctata* and *Lemna minor*	Swine wastewater	27.75 g/m^2^/d (fresh weight)	NA	51	11.2	[68]
5	*Lemna minor*	Domestic wastewater	NA	268.8–1,544.0	30.9–118.9	1.6–25.2	[69]
6	*Lemna gibba*	Domestic wastewater	3.36–13.37 g/m^2^/d (dry weight)	749–871	26.3–29.6	5.2–6.2	[70]
7	*Lemna minor*	Lake reservoir water	NA	NA	5.9	0.25	[71]
8	*Lemna minor*	Industrial Wastewater	NA	544	36.8	1.5	[72]
9	*Lemna gibba*	Industrial drainage water	10.25 kg/m^2^/d (dry weight)	131.2	14.6	2.9	[73]

*NA* Not available, *COD* Chemical oxygen demand, *N* Nitrogen, *P* Phosphorous

Nutritional content in feed water, specifically nitrogen and phosphorus content, play a decisive role in determining the yield and nutritional content in the harvested duckweed biomass [74]. Total nitrogen concentrations ranging from 3.5 to 40 mg N/L, with nitrogen-to-phosphorus ratio of 15:1 has been reported to give optimum duckweed biomass yield [36, 75]. Nitrogen concentrations around 28 mg/L is reported as the optimum nitrogen concentration for prosperous duckweed growth, while nitrogen concentration exceeding 60 mg/L was found to exert substantial toxicity [76]. Ammoniacal form of nitrogen is preferred over nitrates for duckweed growth primarily because of the ease of conversion of ammonia into amino acids [77]. Ammonia is also a limiting nutrient for duckweed growth with greater than 8–12 mg NH_3_-N/L proving to be detrimental for duckweed growth [36]. Acidic to neutral pH of the water (3.0–7.5), temperature ranging from 6°C–33°C and optimal daily light integral between 5 and 20 mol/m^2^ is recommended for enhanced nutrient adsorption by duckweed, indirectly contributing to improved biomass production [75]. Yield as high as 33 tons dry matter/hectare/8 months has been reported from duckweed cultivation in domestic wastewater [70], while industrial wastewater was found to yield 102.5 t/ha/d (dry weight) [73]. Use of wastewater for duckweed growth is instrumental in reducing the cost of production and makes the use of duckweed as soybean substitute competitive, economical and sustainable.

### Contribution to sustainable and local feed systems

Duckweed offers a local, climate-resilient, and low-input alternative in areas where traditional feed ingredients are expensive or scarce. Its use contributes to food and feed security—a key concern in both developing and developed countries. The level of nutritional control possible with duckweed is not typically possible with conventional feed crops like soybean, which are more reliant on fixed soil and climate conditions. Partial replacement of conventional poultry protein meals with duckweed can also assist to recycle nutrients and reduce waste in integrated farming systems further supporting sustainability.

## Challenges, limitations and future directions

**Fig. 2:**
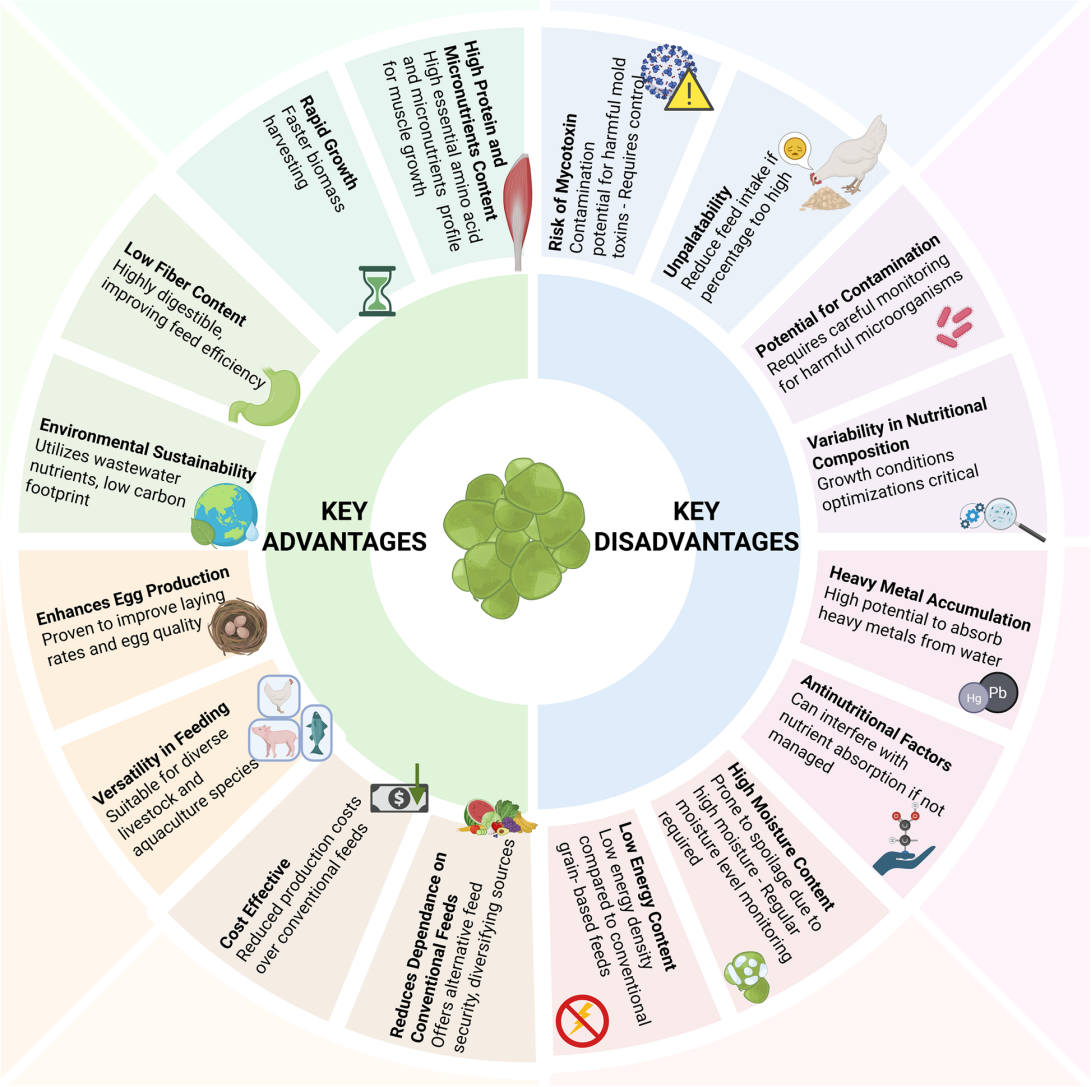
Advantages and disadvantages (limitations) in the use of duckweed as poultry feed supplement (Created in https://BioRender.com)

While duckweed has emerged as a low-cost protein source and is touted as an alternative protein for poultry feed, its practical application is not without challenges and limitations (Fig. 2).

Despite encouraging experimental results, widespread adoption is still constrained by several biological, technical, and economic limitations. Issues such as variability in nutrient content, presence of anti-nutritional factors, scalability of production, and regulatory concerns pose significant hurdles. Moreover, inconsistencies in inclusion rates and lack of standardized processing techniques further complicates its integration into commercial poultry feed systems.

### Anti-nutritional factors (ANFs) and their mitigation

Like other plant-based food, duckweed biomass also contains some ANFs that can limit its use. ANFs and phytochemicals like oxalates, phytic acids, and tannins interfere with nutrient absorption, including calcium and phosphorus (P), and pose difficulty in digestion [1]. High levels of phytates can lead to lower biological phosphorus utilisation as about 70% of phosphorus in plants exists in the form of phytate-P [78]. This indigestibility can be overcome by the supplementation of duckweed with enzymes such as phytase that can increase the feed conversion ratio in the poultry, enhancing the digestibility and acceptance of the duckweed supplemented poultry feed.

Digestive enzymes such as amylases, cellulases and non-digestive enzymes such as phytases, glucose oxidase, and lysozymes have been incorporated in fish and poultry feed to increase the bioavailability of nutrients and increase the efficiency of feed [79]. However, studies on the hydrolysis of duckweed are limited. Enzymatic hydrolysis of duckweed with hydrolytic enzymes Alcalase and Flavourzyme used at concentrations from 0.5% to 3.5% (v/w) increased protein recovery and antioxidant activity of the hydrolysed duckweed [80]. Enzymatic hydrolysis of duckweed proteins by pepsin, chymotrypsin, papain, and trypsin led to higher recovery of bioactive peptides with antihypertensive activity [81]. Fortification of duckweed supplemented feed with diverse enzymes will further aid in enhanced digestibility and therapeutic value of duckweed supplemented poultry feed.

Another promising strategy to increase the digestibility of duckweed is to ferment the duckweed biomass, leading to changes in the nutritional components, thereby making the feed easily digestible. Fermented duckweed has been shown to be a superior feed in combination with *Bacillus* probiotic, enhancing the gut as well as secondary metabolite phenolic compounds, flavonoids, and other bioactive molecules that may contribute to immune system modulation and protection against pathogens [33]. Very limited work has been done on the use of fermented duckweed for poultry diet. Studies have shown that duckweed supplemented with probiotic *Bacillus* strain such as *B. subtilis* and *B. amyloliquefaciens,* improved and supported beneficial gut microbiota and enhanced the intestinal health of poultry. Fermentation of *Lemna minor* and *Spirodela polyrhiza* with these *Bacillus* strains significantly increased the viable probiotic count. The effect of fermentation on duckweed is also dependent on the type of species, as was indicated by higher growth of fermenting organisms on *Lemna minor* as compared to *Spirodela polyrhiza* [82].

In one study, two microorganisms, *Trichoderma* sp. and *S. cerevisiae* were successfully used to ferment the duckweed leading to reduction in the crude fibre [83]. Similar effects were seen when *Lactobacillus casei* and *Saccharomyces cerevisiae* were used to ferment *Lemna minor*. Fermentation increased the protein content, while crude fibre and ash decreased slightly after fermentation [84]. In another study, duckweed fermented using *Bacillus* sp. was incorporated in the diet of rohu fish fingerlings (upto 30%). A significant reduction in tannin (from 1.0% to 0.02%) and phytic acid (from 1.23% to 0.09%) levels and improved growth performance was observed, indicating that fermentation effectively reduces ANFs and enhances the nutritional value of duckweed with no significant change in crude protein [85]. While direct in vivo gut health studies in poultry are still lacking, related research has reported that these probiotic-supplemented feeds enhance feed conversion, pathogen resistance, and nutritional bioavailability. Additionally, these fermented duckweed with lower ANFs have the potential to be incorporated at the higher concentrations in the poultry feed, increasing the overall protein content.

### Large-scale processing and storage concerns

Duckweed biomass contains 95% moisture that poses the risk of emergence of fungal contamination upon long term storage. Monitoring of moisture content hence becomes a critical factor in the long term storage of duckweed biomass. One strategy that has shown promising results to prolong the shelf life is to sundry the biomass, microwave at 900 W and blanch at 100 °C for 3 min. This treatment not only increased the shelf life but also improved the nutrient extraction efficiency [66]. Exploring new methods to harvest, dry and process duckweed to retain maximum nutritional values while ensuring long shelf life requires additional technological interventions and study.

### Genetic improvements for nutritional enhancement

Duckweed is a promising, readily available, and reliable form of protein source to address the needs of the growing population and meet increasing feed demand. Enhanced genetic engineering tools and metabolic engineering can be applied to tailor the metabolic pathway of duckweed for increased amino acid levels and decreased antinutritional factors [33]. Multi-omics integration of duckweed by combining genomics, transcriptomics, proteomics, and metabolomics could provide insights into gene functions related to nutrient uptake and biosynthesis,​ and biofortification of duckweed by introducing genes that enhance micronutrients such as iron, zinc and omega-3 fatty acids, that will increase the nutritional value of duckweed [86]. A vital point of intervention can be Rubisco. Ribulose-1,5-bisphosphate (RuBP) carboxylase/oxygenase (Rubisco) is a plant protein critical for carbon fixation. Rubisco activity is affected by the presence of certain phosphorylated compounds that can act as inhibitors, limiting the activity of Rubisco. Compounds such as CA1P phosphatase (CA1Pase) and XuBP phosphatase can inhibit Rubisco inhibitors, to reactivate Rubisco [87]. Modulation of activity of CA1P phosphatase in duckweed is hence a potential intervention to increase the carbon capture potential of duckweed and increase plant biomass. Another interesting area to explore is the use of Clustered Regularly Interspaced Short Palindromic Repeats (CRISPR) technology. CRISPR-based genome editing to enhance nitrogen assimilation genes or target essential amino acid biosynthetic pathways can impact duckweed amino acid profile, making it more wholesome and rich [88]. Genetically modified duckweed is an unexplored domain that needs more research.

### Nutritional enhancements through cultivation techniques

Dependency of nutritional profile of duckweed on the growth water cannot be overruled or ignored. Conditions of stress, such as nutrient depletion, induces starch accumulation in the plant and insufficient CO_2_ supply could hamper the productivity and quality of duckweed [1]. Hence formulating and maintaining a stable nutrient profile of water is imperative for a constant duckweed harvest. Nutritional content of duckweed alters significantly with the cultivation conditions, including salinity, nutrient availability, temperature, light intensity and the photoperiod. Recent studies focus on the feasibility of manipulating growth conditions (light, nutrients, pH) to optimize protein and amino acid content in duckweed. This makes duckweed a customizable feed ingredient—a factor not easily achievable with conventional plant proteins [25]. Unlike most conventional plant protein sources, whose nutrient profiles are largely fixed by species and soil conditions, duckweed's protein and amino acid content can be significantly altered by adjusting environmental variables during its growth. Prominent among the determinants are light intensity and duration, nutrient availability and water chemistry, especially nitrogen, phosphorous, and potassium concentrations [68]. Additionally, polycultures of duckweeds enhance the immunity of the plants, enabling enhanced nutrient efficiency and higher protein content of the harvested biomass [89]. Since light plays a critical role in plant growth, optimizing light exposure intensity and period can enhance productivity and increase the total protein yield. High intensity of light can shift the production from protein to carbohydrate, thereby necessitating the significance of light in duckweed protein profile.

Tailoring N:P ratio in the growth water causes tailoring of amino acid profile to suit specific feed needs [57]. This targeted cultivation however, although beneficial, is more suited for biomass targeted for human consumption rather than a strategy suited for poultry feed sector. Additionally, use of low nitrogen water can be compensated by the use of inoculum of nitrogen fixing bacteria, such as *Azotobacter*, increasing the nitrogen content in water for enhanced duckweed growth [90]. The fact that *Azotobacter* has probiotic properties will be an added advantage and can make the harvested biomass more wholesome. In addition, inoculum of duckweed ponds with B_12_ producing bacteria will enable a harvest of a duckweed biomass rich in Vit B_12_, another nutrient of high importance in poultry growth and immunity [91]. This underscores the importance of microbiological interventions to enhance the nutritional profile of the harvested duckweed biomass.

### Comprehensive long-term feeding trials to validate benefits

Long-term feeding trials are needed to evaluate the impact of duckweed use on different livestock species, particularly when duckweed is replacing conventional feed sources like soybean meal​ [1]. Unlike soybean meal or fish meal, the optimal inclusion levels of duckweed in poultry diets, its effects on growth, immunity, gut health, and egg quality across different breeds, are still being actively studied. Although previous studies indicate a positive impact, long term trials are limited. This gives researchers and nutritionists a wide scope for further research and innovation.

### Integration with wastewater treatment

One of the most innovative aspects is duckweed’s dual role, its ability to remediate nutrient-rich wastewater (from agriculture or aquaculture) while producing a high-protein biomass. This opens up exciting possibilities for circular farming systems, reduced environmental pollution and improved resource efficiency. Growing duckweed in nutrient-rich agricultural or aquaculture wastewater provides a low-cost, eco-friendly way to produce protein-rich biomass, while simultaneously treating the wastewater. This integrated approach supports both environmental sustainability and feed security. Hence the use of duckweed in poultry feed is a multifunctional, eco-friendly, and locally adaptable solution in the era of climate change, soil degradation and resource depletion [92].

## Conclusion

There is a need in the poultry feed sector to meet the growing demand of poultry feed in line with changing climatic conditions. Duckweed is a fast growing aquatic plant with high protein content and a diverse nutritional profile, making it a promising source of alternate protein for poultry feed. The ease of cultivation on wastewater makes it a sustainable low-cost feed additive. Although duckweed has a high amount of crude protein, the compatibility of the protein with metabolic system of different poultry remains a challenge. Improvement of protein content of plants through genetic engineering, comprehensive feed trails and optimization of duckweed nutritional profile are promising future lines of work to increase the acceptability and integration of duckweed in poultry feed sector. Research on alternate and regionally adapted poultry feed sources—such as duckweed—can help ensure feed security, reduce greenhouse gas emissions, and promote circular bioeconomy practices in the poultry sector. This not only enhances poultry productivity and animal health, but also supports economic stability for farmers while minimizing environmental footprints. Ultimately, diversified feed strategies strengthen food security and makes poultry production more resilient to the growing challenges of climate change.

## Data Availability

The datasets used and/or analysed during the current study are available from the corresponding author on reasonable request.

## Abbreviations

ANF: Anti-nutritional factor
COD: Chemical oxygen demand
DDGS: Distillers dried grains with solubles
EE: Ether extract
FCR: Feed conversion ratio
